# Publisher Correction: Causes of model dry and warm bias over central U.S. and impact on climate projections

**DOI:** 10.1038/s41467-017-02011-3

**Published:** 2018-01-11

**Authors:** Yanluan Lin, Wenhao Dong, Minghua Zhang, Yuanyu Xie, Wei Xue, Jianbin Huang, Yong Luo

**Affiliations:** 1 Ministry of Education Key Laboratory for Earth System Modeling, Department of Earth System Science, and Joint Center for Global Change Studies (JCGCS), Tsinghua University, Beijing, 100084 China; 2 School of Marine and Atmospheric Sciences, Stony Brook University, Stony Brook, NY 11794-5000 USA; 3 Institute of Atmospheric Physics, Chinese Academy of Sciences, Beijing, 100029 China; 4 Department of Computer Science and Technology, Tsinghua University, Beijing, 100084 China

*Nature Communications* **8**:881 10.1038/s41467-017-01040-2; Article published online: 12 Oct 2017

The original version of this Article contained an error in Figure 2. In panel a, the *x* axis of the graph was incorrectly labeled ‘precipitation bias’, and should have read ‘negative precipitation bias’. This error has been corrected in both the PDF and HTML versions of the Article.

