# Supplementary material for: Causes of model dry and warm bias over central U.S. and impact on climate projections
Source: Nat Commun. 2017 Oct 12;8:881. doi: 10.1038/s41467-017-01040-2 (PMC5638845; doi:10.1038/s41467-017-01040-2)
Supplement: Supplementary file 1 — Supplementary Information [file 41467_2017_1040_MOESM1_ESM.pdf]

Supplementary information

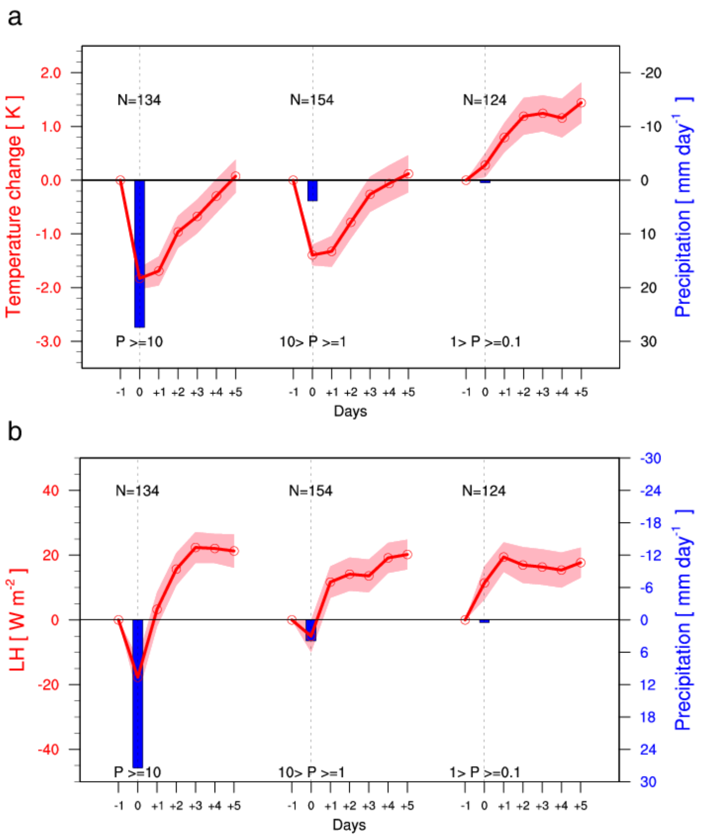

Supplementary Figure 1. Cooling effects. (**a**) Relative change of temperature (K, left *y*-axis) and (**b**) relative change of net downward radiant heat flux (W m^{-2}, left *y*-axis) after three different intensities of rainfall events (mm day^{-1}, right *y*-axis) within a week. The temperature and net downward radiant heat flux changes are calculated with respect to the day prior to the rainfall event. Numbers *N* indicate the sample sizes in each rainfall categories.

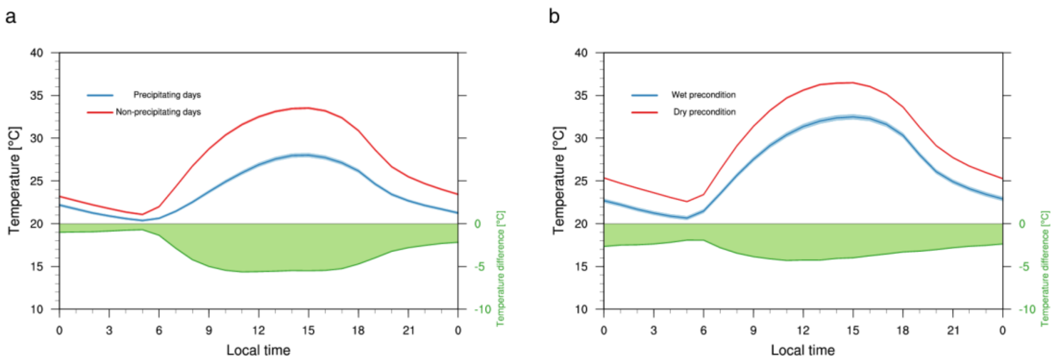

Supplementary Figure 2 | Composite surface temperature diurnal variations. (**a**) Diurnal cycle of surface temperature averaged over precipitating days and non-precipitating days. (**b**) Diurnal cycle of surface temperature averaged over non-precipitating days with wet precondition and non-precipitating days with dry precondition. The sample sizes in **a** are 288 and 945 while they are 49 and 73 in **b**. Shading indicates one s.e.m. for each composite. The green areas below are their respective differences.

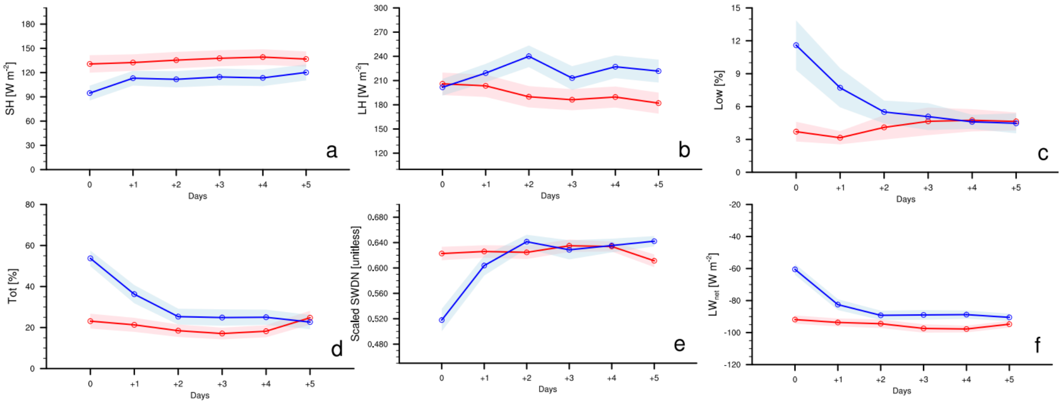

Supplementary Figure 3. Evolution of (**a**) sensible heat flux, (**b**) latent heat flux, (**c**) low cloud, (**d**) total cloud, (**e**) scaled downward shortwave radiation, and (**f**) net longwave radiation under wet (blue) and dry (red) preconditions. The scaled downward shortwave radiation is the ratio of downward shortwave radiation near the surface to that at the top of atmosphere. Shading indicates one s.e.m. for each composite. Sample sizes for wet and dry preconditions are 49 and 73, respectively.

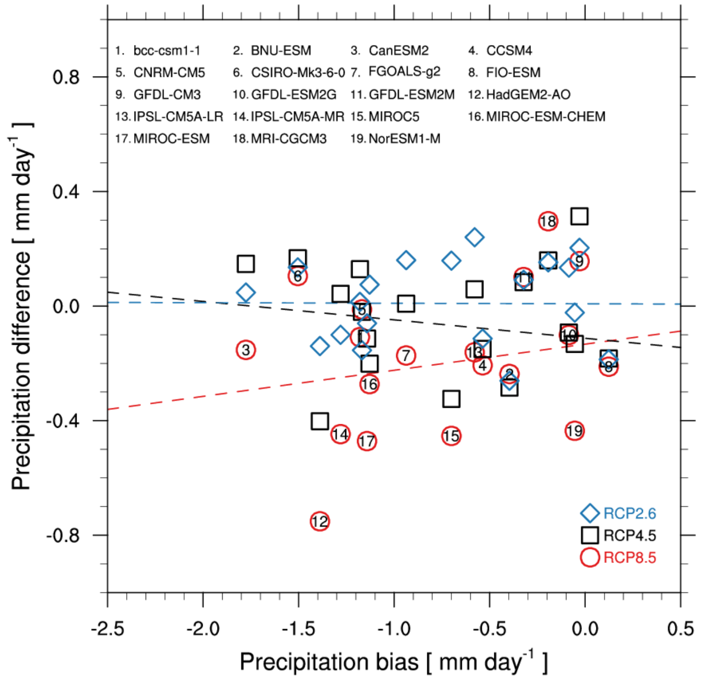

Supplementary Figure 4. Precipitation correspondence. The relationships between precipitation changes (2080-2099 relative to 1981-2000) and precipitation bias for the three scenarios. RCP2.6, RCP4.5, and RCP8.5 are represented by blue, green, and red symbols. Dash lines indicate the linear fit to each scenario. Numbers with circle represent the correspondent models. The regression equations for the three RCP scenarios are: $\delta P = 0.02P_{bias} + 0.04\ (r^2 = 0.01;\ P > 0.6)$, $\delta P = -0.01P_{bias} - 0.05\ (r^2 = 0.0;\ P > 0.8)$, and $\delta P = 0.13P_{bias} + 0.09\ (r^2 = 0.06;\ P > 0.1)$, respectively.

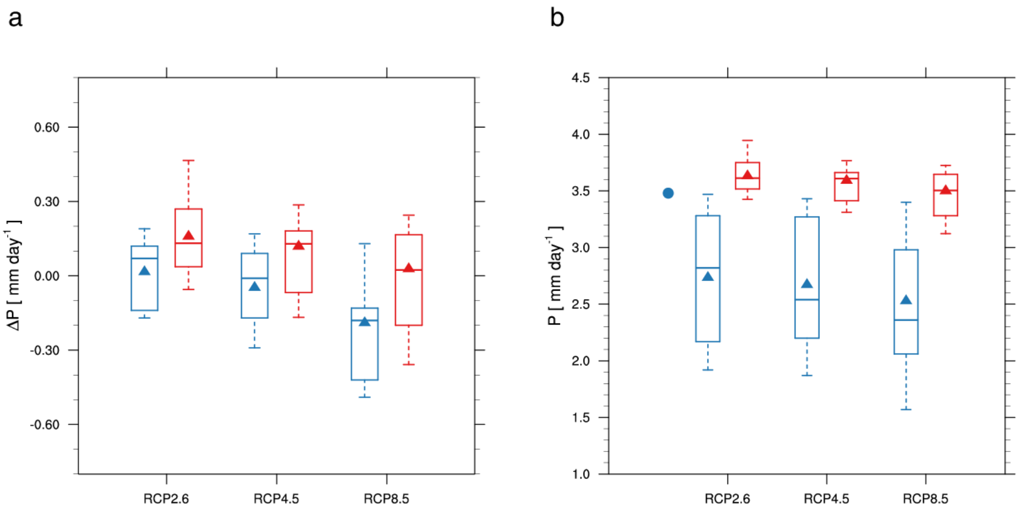

Supplementary Figure 5. Bias correction for precipitation. (**a**) Boxplot of precipitation changes with (red)/without (blue) bias correction for the three scenarios. The bias correction is based on the correspondence between precipitation bias and temperature bias shown in Fig. 2a. (**b**) As in **a** but for absolute precipitation. Solid dots in the first columns represent the observations (averaged over 1980-1999). The boxes indicate 10%-quantile, 25%-quantile, median, 75%-quantile, and 90%-quantile, respectively. Solid triangles stand for the respective multi-model mean.

Supplementary Table 1. List of included CMIP models, their original resolution, and bias of precipitation and temperature averaged over central U.S.

CMIP5 models	Model Resolution	Bias (Annual)		Bias (Summer)	
		Precipitation (mm/d)	Temperature (K)	Precipitation (mm/d)	Temperature (K)
bcc-csm1-1	128x64	-0.88	0.22	-1.16	2.10
BNU-ESM	128x64	-0.29	0.10	-0.37	2.23
CanESM2	128x64	-0.94	4.43	-1.82	6.58
CCSM4	288x192	-0.60	1.03	-0.54	2.54
CNRM-CM5	256x128	-0.80	0.81	-1.18	2.03
CSIRO-MK3-6-0	192x96	-0.69	0.95	-1.47	5.50
FGOALS-g2	128x60	-0.70	-3.26	-0.94	-1.42
FIO-ESM	128x64	-0.34	0.97	0.12	0.03
GFDL-CM3	144x90	-0.17	-0.78	0.01	-1.91
GFDL-ESM2G	144x90	-0.43	-1.05	-0.09	-0.55
GFDL-ESM2M	144x90	-0.35	-0.38	-0.24	0.11
HadGEM2-AO	192x145	-0.42	0.89	-1.41	3.94
IPSL-CM5A-LR	96x96	-0.75	-0.25	-0.59	0.44
IPSL-CM5A-MR	144x143	-0.88	0.60	-1.28	2.30
MIROC5	256x128	-0.40	1.88	-0.73	3.40
MIROC-ESM-CHEM	128x64	-0.83	2.58	-1.08	3.76
MIROC-ESM	128x64	-0.79	2.81	-1.18	3.95
MRI-CGCM3	320x160	-0.09	-0.88	-0.20	-0.66
NorESM1-M	144x96	-0.57	-0.52	-0.06	-0.27
Multi-model mean		-0.57	0.53	-0.75	1.79

Supplementary Table 2. Changes in precipitation, temperature, cloud fraction, sensible heat, latent heat, and radiation terms between three different rainfall intensities and non-precipitating days. The precipitation is averaged between 0000 and 0600 Local Solar Time while the other variables are averaged between 0600 and 1800 Local Solar Time. The numbers in the parentheses indicated the sample size.

	ΔP (mm/d)	ΔT (°C)	ΔCloud fraction (%)		ΔLH (W/m^2)	ΔSH (W/m^2)	ΔSW (W/m2)		ΔLW (W/m2)	
			3 types	Total			Upward& downward	Net	Upward& downward	Net
Large precipitation (134)	42.42	-5.6	3.30 (low)<br>4.17 (mid)<br>27.75 (high)	35.03	-42.85	-47.99	-160.69 (downward)<br>-37.11 (upward)	-123.58	-1.62 (downward)<br>-38.51 (upward)	36.89
Moderate precipitation (154)	4.87	-4.09	3.99 (low)<br>6.05 (mid)<br>21.47 (high)	31.63	-33.68	-25.95	-121.06 (downward)<br>-28.65 (upward)	-92.41	-0.25 (downward)<br>-28.55 (upward)	28.30
Light precipitation (124)	0.72	-2.73	2.67 (low)<br>3.50 (mid)<br>7.08 (high)	13.29	-2.6	-16.75	-54.99 (downward)<br>-13.66 (upward)	-41.33	-3.94 (downward)<br>-19.63 (upward)	15.69

Note: The total cloud fraction is not equal to the sum of three different types of cloud due to the overlap

Supplementary Table 3. Contrast of solar insolation at the top of atmosphere, surface temperature, cloud fraction, sensible heat, latent heat, and radiation terms between wet and dry preconditions. All the variables are averaged between 0600 and 1800 Local Solar Time on the following 5-consecutive non-precipitating days. The radiant and turbulent fluxes are positive downward. The numbers in the parentheses indicated the sample size.

	$SWDN_{TOA}$ (W/m2)	T (℃)	Cloud fraction (%)		LH (W/m^2)	SH (W/m^2)	SW (W/m2)		LW (W/m2)	
			3 types	Total			Upward& downward	Net	Upward& downward	Net
Wet precondition (49)	847.23	30.10	5.25 (low)<br>6.83 (mid)<br>14.11 (high)	26.54	-223.49	-108.46	554.25 (downward)<br>-113.43 (upward)	440.82	402.43 (downward)<br>-489.43 (upward)	-87.00
Dry precondition (73)	828.53	31.73	4.68 (low)<br>5.50 (mid)<br>13.29 (high)	23.79	-207.00	-115.80	544.12 (downward)<br>-116.12 (upward)	428.00	411.33 (downward)<br>-499.47 (upward)	-88.14
Wet precondition minus Dry precondition	18.70	-1.63	0.57 (low)<br>1.33 (mid)<br>0.82 (high)	2.75	-16.49	7.34	10.13 (downward)<br>-2.69 (upward)	12.82	-8.90 (downward)<br>-10.04 (upward)	1.14

Note: The total cloud fraction is not equal to the sum of three different types of cloud due to the overlap

Supplementary Table 4. List of projected precipitation and temperature changes for the three RCP scenarios over central U.S. from 19 CMIP5 models.

CMIP5 models	RCP2.6		RCP4.5		RCP8.5	
	Precipitation (mm/d)	Temperature (K)	Precipitation (mm/d)	Temperature (K)	Precipitation (mm/d)	Temperature (K)
bcc-csm1-1	-0.02	1.45	0.09	2.12	-0.14	5.49
BNU-ESM	-0.22	2.44	-0.25	3.66	-0.20	6.44
CanESM2	0.07	2.12	0.17	2.89	-0.13	6.61
CCSM4	-0.12	1.49	-0.16	2.47	-0.21	5.19
CNRM-CM5	-0.14	2.15	-0.01	2.79	0.00	5.12
CSIRO-MK3-6-0	0.12	2.63	0.15	3.75	0.09	6.41
FGOALS-g2	0.15	1.26	0.00	2.43	-0.18	5.46
FIO-ESM	-0.17	0.09	-0.17	1.15	-0.20	4.08
GFDL-CM3	0.20	3.81	0.31	4.83	0.16	7.89
GFDL-ESM2G	0.11	0.74	-0.12	2.05	-0.13	4.34
GFDL-ESM2M	0.12	0.44	0.11	1.45	0.13	3.84
HadGEM2-AO	-0.17	1.93	-0.43	5.02	-0.78	7.20
IPSL-CM5A-LR	0.23	1.35	0.05	3.03	-0.17	6.45
IPSL-CM5A-MR	-0.14	1.86	0.00	3.05	-0.49	7.34
MIROC5	0.19	1.79	-0.29	3.76	-0.42	6.26
MIROC-ESM-CHEM	0.10	2.92	-0.18	4.71	-0.25	7.97
MIROC-ESM	-0.04	3.09	-0.10	4.49	-0.45	8.52
MRI-CGCM3	0.19	0.98	0.20	1.88	0.33	3.80
NorESM1-M	-0.03	1.64	-0.14	3.24	-0.44	5.98
Multi-model mean	0.02	1.80	-0.04	3.09	-0.18	6.02
